# Radon emission from soil gases in the active fault zones in the Capital of China and its environmental effects

**DOI:** 10.1038/s41598-018-35262-1

**Published:** 2018-11-13

**Authors:** Zhi Chen, Ying Li, Zhaofei Liu, Jiang Wang, Xiaocheng Zhou, Jianguo Du

**Affiliations:** 1 CEA Key Laboratory of Earthquake Prediction (Institute of Earthquake Forecasting), China Earthquake Administration, Beijing, 100036 China; 2 Hebei Earthquake Agency, Shijiazhuang, 050022 China

## Abstract

The release of radon in active fault zones is a sustained radioactive pollution source of the atmospheric environment. The species, concentration and flux of radon emitted in soil gas in active fault zones in the Capital of China were investigated by *in-situ* field measurements. Two main species of radon discharging from soil gas in active fault zones were identified, including radon diffused and dispersed from permeable soil, and upwelling from faults. Higher concentrations and flux of radon from faults were observed in the Bohai Bay Basin due to the accumulated uranium in the sandstone reservoirs and higher permeability of the strata and bed rocks. Increased radon released by strong earthquakes persists, with the max flux of 334.56 mBq m^−2^ s^−1^ observed in FN (Fengnan district) located at the epicenter of the 28 July, 1976 Tangshan *M*_*S*_ 7.8 earthquake. The level of radon released in 8 of 22 locations within the Basin and Range Province (to the west of Taihangshan piedmont fault Zone) reached level 2, and 13 of 14 locations within the Bohai Bay Basin reached levels 2–4, according to the Chinese Code (GB 50325–2001, 2006). Corresponding protective and safety measures should be in place to protect the health of nearby residents, due to their exposure to radon emitted from the faults. Also, the concentration of radon in active fault zones should be investigated to assess the possible risk, before land-use is planned.

## Introduction

Radon (from here on referred to as ^222^Rn) is a naturally occurring, odorless, colorless and radioactive noble gas which has a half-life of 3.82 days. It is a by-product of the naturally occurring radioactive decay of radium in the ^238^U decay series formed in the crust^1^. Radon is ubiquitous outdoors and indoors, and can decay into a number of short-lived products (progeny) that are also radioactive. Both elemental and bound radon progenies can deposit in the lungs when inhaled and irradiate lung tissue as decay continues. As a result, radon has been identified as the second leading cause of lung cancer, the first being smoking. Statistically, approximately 11,000 lung cancer patients die each year in the USA and approximately 55,000 in China, as a result of radon exposure^2–4^. Consequently, the associated health risks resulting from the exposure to radon has received increasing attention in recent years^5,6^. According to the 2013/59/EURATOM Council Directive of the European Union^7^, Member States are required to establish a national action plan addressing the long-term risks from radon exposure for any source of radon, whether it be from the soil, building materials or water.

Faults and fractures are preferential migration pathways for radon gas and carrier gases (CO_2_, N_2_, etc.), from the deep layers of the crust to the surface, due to their greater permeability and porosity compared to surrounding rock, enabling gases to buoyantly migrate upwards. Radon gas discharged through faults and fractures in active fault zones can be enhanced by fault and earthquake activity^8–15^. Radon concentration and flux surveys along active fault zones have been performed for earthquake research and prediction, and high soil radon concentrations and fluxes are often reported in active fault zones worldwide^16–23^. The soil radon concentration and fluxes observed along the southeastern section of the Haiyuan fault are between 1.0~38.3 kBq m^−3^ and 5.2~828.6 mBq m^−2^ s^−1^ ^24^ and for the Ravne fault in NW Slovenia are between 0.9~32.9 kBq m^−3^ and 1.1~41.9 mBq m^−2^ s^−1^ ^25^. The maximum soil radon concentration and flux from the rupture zones produced by the 2008 Wenchuan *M*_*S*_ 8.0 earthquake in Western Sichuan, China, was 106.6 kBq m^−3^ and 1976.0 mBq m^−2^ s^−1 ^^26^, suggesting that the release of radon gas through faults and fractures in seismic zones could result in a health risk to people living in the adjacent areas along fault zones, according to Chinese Code for indoor environmental pollution control of civil building engineering^26–30^.

This study focuses on the characteristics and environmental effects of radon release from active fault zones in seismic zones near Beijing, China.

**Figure 1:**
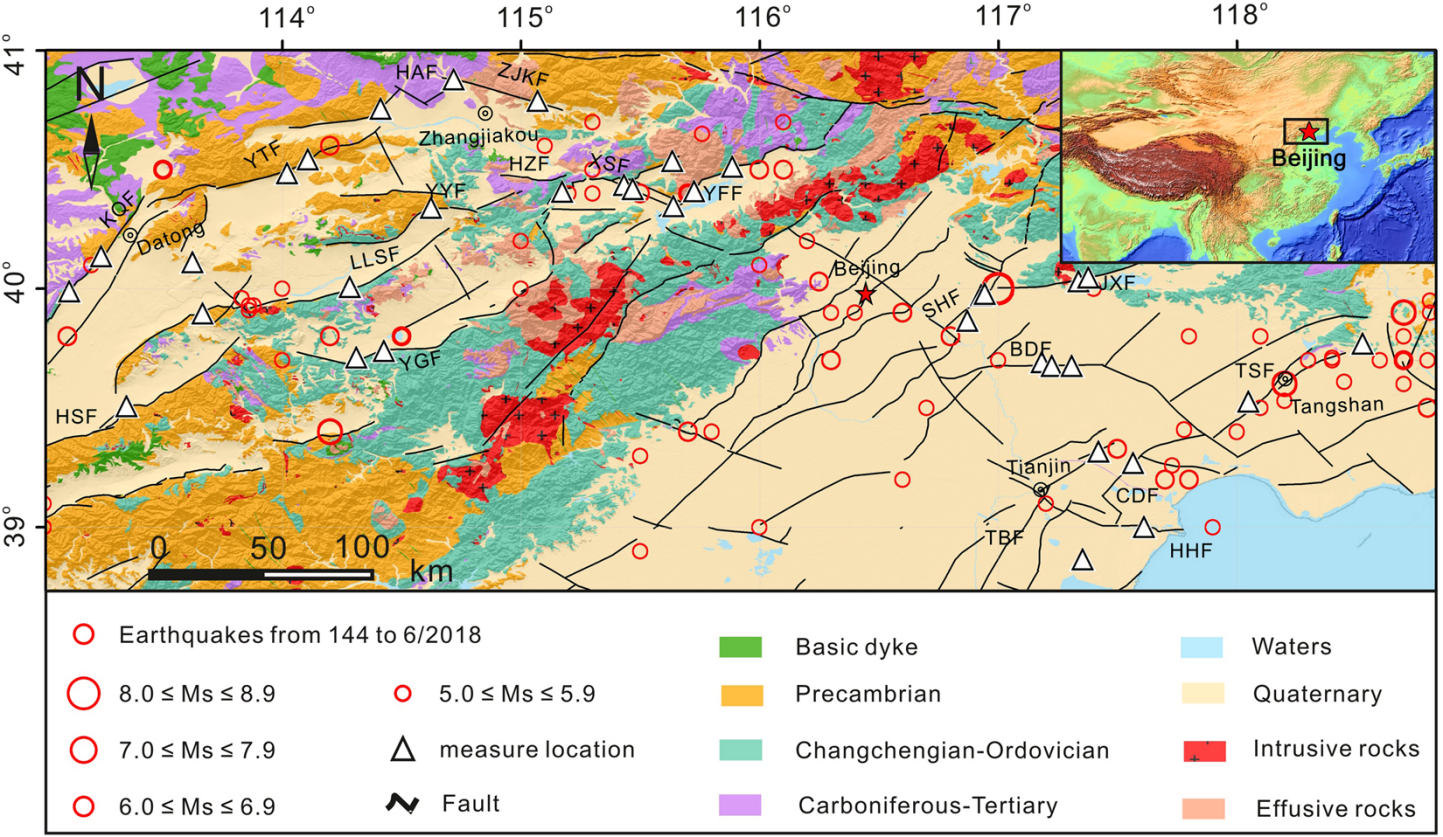
Schematic geologic map of the research area. Inset map shows location of the studied region in China.

The capital is in the north of northern China and belongs to the Trans-North China Block and Eastern Block (Fig. 1). The tectonic framework, morphology and topography of the study area were formed as a result of three stages of tectonic evolution: the formation of crystalline basement in the Precambrian Era, the development of platform sedimentary cover from late Precambrian to Paleozoic Eras and the Crust activation from the Mesozoic to Cenozoic Eras^31^. The tectonic setting in the area is complex, demarcated by the Taihangshan piedmont fault zone, the basin and range tectonics zone distributed to the west, and the Bohai Bay basin to the east. The Precambrian metamorphic rocks, Carboniferous-Tertiary sandstone, Changchengian-Ordovician carbonates and Quaternary sediments occur in most of the area. The Quaternary sediments occur in the area of the Bohai Bay basin, and intermediate-acid intrusive rocks and intermediate-acid effusive rocks are exposed and are scattered throughout the Taihangshan piedmont fault zone area. In total, 19 active faults oriented in the NE-SW direction occur in the area and were measured in this study, with 12 normal faults stretching to the west of Beijing, and the other 7 strike slip faults to the east. The study area is historically seismically active; 18 great earthquakes (*Ms* > 6.0) have occurred in the area since 1618, including the Sanhe-Pinggu earthquake *M*s = 8.0 in 1679 along the Xiadian fault zone, the Tangshan earthquake *M*_*S*_ = 7.8 in 1976 along the Tangshan fault zone, followed by four aftershocks with magnitudes greater than *M*s = 6.0 (Fig. 1).

## Results

The concentrations and fluxes of radon in the soil gas from the active fault zones in the seismic areas near Beijing, China are listed in Tables 1 and 2.

**Table 1:** Descriptive statistics of soil gas radon concentration in the active fault zones in the Capital of China.

Fault	Location	Times	Mean	Median	Min	Max	UQ	LQ	IQR	SD	T^1^	T^2^	MB	MOF	MOF/MB	Skewness	Kurtosis	ρ-value
			kBq m^−3^															
KQF	SJC	9	10.36	9.24	1.82	27.03	10.93	9.79	1.14	4.89	—	9.28	6.76	14.95	2.06	0.97	0.50	0.00
	SHZ	9	7.86	7.58	1.04	21.56	8.24	7.48	0.77	3.07	—	11.62	7.65	16.61	2.17	1.09	3.00	0.00
HSF	NCY	6	5.82	5.78	2.62	11.97	6.15	5.48	0.68	1.90	—	9.34	5.71	10.63	1.86	0.69	0.34	0.02
LLSF	DHZK	9	4.01	3.83	0.89	9.07	4.19	3.83	0.36	1.45	—	7.32	4.00	7.65	1.91	0.46	0.05	0.01
	YJY	5	5.13	4.74	0.46	15.70	5.64	4.62	1.02	2.57	—	10.25	4.88	12.53	2.57	1.15	2.83	0.00
DSF	DTHS	5	7.75	6.96	2.56	17.46	8.46	7.05	1.42	3.50	—	9.37	6.05	11.96	1.98	0.80	0.09	0.00
YTF	YLK	5	5.03	3.92	0.81	22.54	5.64	4.41	1.23	3.06	—	4.44	3.13	8.06	2.58	1.58	3.65	0.00
	ZZK	9	9.17	9.24	0.87	19.56	9.61	8.74	0.87	3.42	—	—	8.99	—	—	−0.21	−0.06	0.13
HAF	YJG	5	6.06	5.88	1.00	20.69	6.53	5.60	0.93	2.59	—	11.81	6.48	16.88	2.61	1.69	6.85	0.00
	ZJY	5	5.13	4.67	0.26	20.69	5.54	4.71	0.84	2.74	3.19	11.85	6.23	16.68	2.84	1.49	4.86	0.00
YYF	NKC	5	10.17	9.70	2.16	23.90	11.22	9.12	2.11	4.85	2.96	17.27	7.86	19.78	2.65	0.78	0.18	0.00
YGF	YXZ	9	7.03	6.37	0.94	21.27	7.44	6.62	0.82	3.33	1.34	12.28	6.61	14.45	2.19	0.74	0.30	0.00
	BKC	5	7.19	5.95	0.63	20.76	8.06	6.33	1.73	4.23	—	10.81	5.68	13.49	2.37	0.99	0.74	0.00
ZJKF	WQX	5	15.96	13.85	5.43	32.70	17.23	14.68	2.55	6.40	—	15.74	9.90	20.06	1.84	0.63	−0.60	0.00
	QBK	5	11.03	9.43	1.85	33.34	12.21	9.85	2.36	5.75	—	20.83	8.74	26.01	2.56	1.33	2.35	0.00
HZF	HJP	9	11.58	10.23	1.41	33.00	12.36	10.81	1.55	6.04	—	18.84	9.23	23.86	2.33	1.05	1.12	0.00
	XHZ	9	5.75	5.39	0.00	21.47	6.15	5.34	0.81	3.19	0.94	11.52	6.54	14.09	2.15	0.62	0.28	0.00
XSF	DYZ	7	9.26	9.16	0.54	23.82	9.99	8.52	1.47	5.11	1.04	18.22	9.98	19.43	2.05	0.21	−0.57	0.01
	LTTC	3	8.36	8.42	0.31	18.22	9.17	7.55	1.62	3.50	—	—	7.12	—	—	0.32	0.81	0.17
YFF	BYC	4	6.26	6.04	0.69	10.60	6.78	5.73	1.06	1.94	—	—	6.24	—	—	−0.16	0.53	0.73
	CYF	7	13.79	12.81	0.87	59.82	14.75	12.83	1.91	6.72	—	27.50	12.70	29.11	2.53	2.11	11.47	0.00
	YHM	5	10.89	9.41	0.20	67.83	12.84	8.94	3.91	9.79	1.01	15.11	9.36	22.91	2.45	2.66	11.63	0.00
SHF	DDG	5	22.93	24.20	1.99	58.40	24.57	21.30	3.26	12.71	—	49.27	26.31	55.48	2.11	0.34	−0.39	0.00
	PGZ	5	23.95	23.38	1.34	71.14	25.49	22.41	3.08	13.29	—	55.00	25.36	59.89	2.36	0.52	−0.04	0.00
	QXZ	5	18.95	17.85	3.20	57.49	20.11	17.79	2.32	9.05	—	26.20	16.47	33.53	2.04	1.01	1.51	0.00
BDF	WJKC	3	31.41	30.49	0.57	71.75	32.67	28.15	6.52	15.39	—	48.93	32.12	64.14	2.00	3.28	10.56	0.00
	BHC	3	18.66	18.04	4.67	42.00	20.26	17.06	3.20	7.55	—	31.00	17.68	38.19	2.16	0.80	0.95	0.01
	CJA	3	30.76	30.78	0.75	67.58	34.01	27.51	6.50	15.34	—	—	30.39	—	—	0.12	−0.51	0.55
JXF	NYC	3	19.80	18.07	0.37	53.51	21.91	17.70	4.21	9.93	—	—	19.59	—	—	0.55	0.55	0.12
	WZ	3	19.38	18.07	0.22	55.85	22.43	16.33	6.10	14.39	—	41.60	17.39	46.57	2.68	0.46	−0.71	0.01
TJBF	ZTD	3	24.17	24.02	0.27	97.55	33.83	15.51	9.32	22.13	—	41.14	28.06	61.65	2.20	1.89	3.01	0.00
CDF	HJW	3	15.71	14.77	0.48	30.39	17.16	14.26	2.90	6.60	—	12.15	12.85	23.29	1.93	2.32	5.10	0.00
	BHD	3	17.71	16.68	0.34	69.20	19.99	15.43	4.56	10.77	—	22.00	18.28	37.28	2.04	3.00	12.44	0.00
HHF	DSG	3	20.33	20.98	0.41	49.78	23.36	17.30	6.05	14.29	—	35.00	18.87	41.00	2.17	0.06	−1.26	0.00
TSF	FN	3	14.97	11.68	0.68	57.67	19.08	10.87	8.21	12.32	2.76	32.73	13.55	52.89	3.90	1.30	2.42	0.00
	WFS	3	14.50	14.78	0.47	32.74	16.88	12.12	4.76	7.63	—	—	13.84	—	—	0.32	−0.14	0.62

UQ: Upper Quartile, LQ: Lower Quartile, IQR: Interquartile range, SD: Standard Deviation, T^1^: the lower threshold in the Q-Q plots, T^2^: the upper threshold in the Q-Q plots, MB: average values of the data between the T^1^ and T^2^, MOF: mean value of fault-origin radon concentration, which was calculated using the average values of the data above T^2^, ρ-value: alpha level or significance level, “−”: no data.

**Table 2:** Descriptive statistics of soil gas radon flux measured in the active fault zones in the Capital of China.

No.	Fault	Location	Times	Number	Mean	Median	Maximum	Minimum
					mBq m^−2^ s^−1^			
F1	KQF	SJC	9	12	67.03	55.03	206.71	8.06
		SHZ	9	12	55.05	51.97	128.43	14.82
F2	HSF	NCY	6	12	25.04	19.27	50.75	0.00
F3	LLSF	DHZK	9	12	29.09	24.18	100.98	6.21
		YJY	5	12	42.95	22.96	116.16	12.55
F4	DTHSF	DTHS	5	12	58.29	44.22	146.81	23.68
F5	YGTZF	YLK	5	12	25.75	15.47	88.66	4.73
		ZZK	9	12	49.59	39.34	142.09	6.27
F6	HAF	YJG	5	12	32.81	29.63	68.91	12.55
		ZJY	5	12	21.41	13.24	50.90	3.45
F7	YYF	NKC	5	12	24.17	16.52	95.30	0.00
F8	YXGLF	YXZ	9	12	31.62	29.40	107.63	3.59
		BKC	5	16	43.08	35.81	114.72	5.47
F9	ZJKF	WQX	5	20	58.46	40.92	201.27	8.68
		QBK	5	20	51.01	38.72	197.46	0.74
F10	HLZLF	HJP	9	20	33.68	24.47	104.49	5.43
		XHZ	9	20	30.60	22.17	119.66	0.00
F11	XBASCF	DYZ	7	20	30.24	18.93	60.80	3.08
		LTTC	3	20	32.50	27.52	63.47	13.41
F12	YQFSF	BYC	4	20	41.21	36.40	93.69	14.30
		CYF	7	20	50.88	41.58	125.43	9.94
		YHM	5	20	43.93	40.68	101.38	12.29
F13	SHF	DDG	5	20	71.92	54.30	138.61	30.19
		PGZ	5	20	93.50	107.81	150.01	29.82
		QXZ	5	20	49.24	42.29	117.49	10.85
F14	BDF	WJKC	3	20	95.24	111.35	174.86	15.56
		BHC	3	24	78.25	76.46	229.99	23.91
		CJA	3	28	86.05	77.73	138.38	37.90
F15	JXF	NYC	3	28	40.69	41.99	94.94	9.45
		WZ	3	36	83.06	81.55	180.47	48.90
F16	TJBF	ZTD	3	36	31.43	24.46	72.85	0.00
F17	CDF	HJW	3	36	57.53	54.12	116.88	30.12
		BHD	3	36	85.15	62.54	159.11	27.62
F18	HHF	DSG	3	36	129.74	53.05	295.74	0.00
F19	TSF	FN	3	36	94.46	36.30	334.56	16.55
		WFS	3	36	54.27	50.78	93.05	22.41

Descriptive statistics (i.e. min and max values, mean, median, standard deviation (SD), interquartile range (IQR), lower interquartile (LQ) and upper interquartile (UQ)), number of repeated measurements and concentration values of radon are listed in Table 1. The minimum and maximum concentrations of radon in the soil gas ranged from 0.20 kBq m^−3^ to 5.43 kBq m^−3^ and 9.07 kBq m^−3^ to 97.55 kBq m^−3^, respectively. The mean concentrations of radon were 4.01 to 31.41 kBqm^−3^, comparable to that of the median concentrations with a range from 3.83 kBqm^−3^ to 30.78 kBq m^−3^. The LQ and UQ for the concentration of radon varied from 4.19 kBq m^−3^ to 34.01 kBq m^−3^ and 3.83 kBq m^−3^ to 28.15 kBq m^−3^, respectively, and the IQR ranged from 0.36 to 9.32 kBq m^−3^. The SD ranged from 1.45 to 22.13 kBq m^−3^, and the ρ-values ranged from 0.00 to 0.73.

The flux of radon (i.e. min and max values, mean, median, the quantity of data and times for repeated measurement) are listed in Table 2. The minimum and maximum flux of radon in the soil gas varied from 0.00 mBq m^−2^ s^−1^ to 48.90 mBq m^−2^ s^−1^ and 50.75 mBq m^−2^ s^−1^ to 334.56 mBq m^−2^ s^−1^, respectively. The mean flux of radon ranged from 21.44 to 129.74 mBq m^−2^ s^−1^, which is comparable to that of the median flux ranging from 13.24 mBq m^−2^ s^−1^ to 111.35 mBq m^−2^ s^−1^.

## Discussion

**Figure 2:**
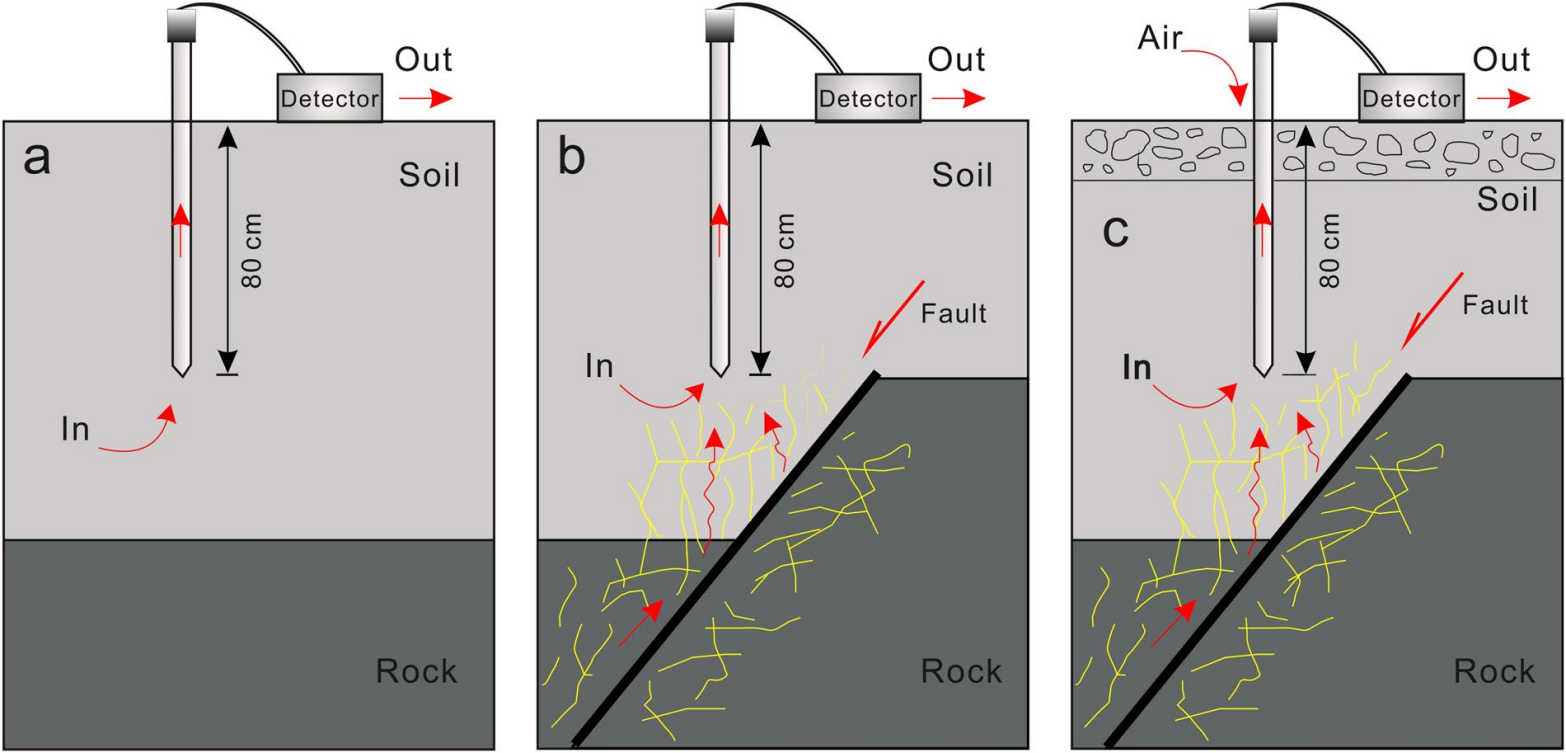
A map of different origins of soil gas radon observed in the profiles across the fracture zones, the yellow line indicates the fractures produced by activity of the fault.

Radon, a by-product of the natural radioactive decay of ^238^U, occurs widely in soil and rock^32^. It can escape upwards to the shallow crust by diffusing and dispersing in permeable soils, or by migration upward along preferential pathways, such as fractures and faults^33,34^. Therefore, radon observed along the gas profiles across active faults is primarily from two origins: (1) the radon diffuses and disperses in the soil, generated by the decay of ^238^U accumulated in the soil (Fig. 2a), (2) the radon migrates upward through the faults and fractures transferred by carrier gases (CO_2_, N_2_, etc.) (Fig. 2b), which can be produced by the decay of ^238^U from the deeper crust and in the CaSiO_3_ perovskite phase in the mantle^26,35^. Due to the diffusion coefficient in dry soil (5 × 10^−6^ m^2 ^s^−1^) and half-life (3.82 days) of radon^36^, the detectable distance for radon diffusing and dispersing in soil is within several metres^37^. The radon concentration in superficial soil is usually low, thousands of Bq m^−3^, and is subject to dilution by air with extremely low radon concentration, several Bq m^−3^ ^32^. Faults are the preferred and fastest pathway for the uprising of gases from deep within earth^13,38^, enabling the escape of gaseous radon generated from the decay of ^238^U in rocks deep within the crust to the soil surface^39,40^. This is very efficient in the presence of carrier gases (CO_2_, N_2_, etc.), and results in high radon concentration in active fault zones^41,42^. High radon concentrations in the range of 20 to 80 kBq m^−3^ are reported in active fault zones worldwide^21,23,35^. Therefore, the upward migrating radon through faults is usually at a higher concentration than radon diffusing and dispersing in the soil surface.

**Figure 3:**
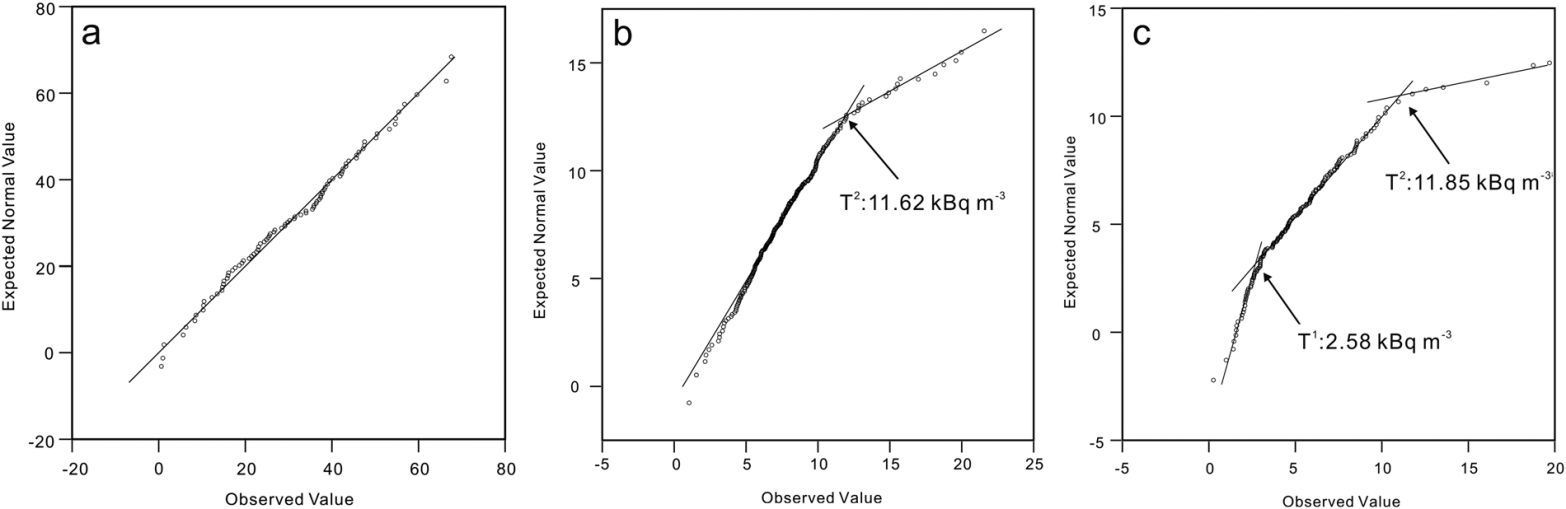
Quantile-quantile plots (Q-Q plots) of the concentration of radon at CJA (**a**), SJC (**b**), and ZJY (**c**).

A radon origin distinction analysis was carried out using the Kurtosis-Skewness test and Q-Q (quantile-quantile) plots. All the ρ-values of the concentration of radon measured between 2012 and 2016 were < 0.05, with the exception of 6 locations (ZZK, LTTC, BYC, CJA, NYC and WFS) (Table 1), indicating that the radon concentrations were mostly non-normal distribution. The Q-Q plots of radon concentration from 36 locations show single linear distribution patterns (Fig. 3a, ZZK, LTTC, BYC, CJA, NYC and WFS), 3 linear segments (Fig. 3c, ZJY, YXZ, DYZ, NKC, XHZ, YHM and FN) and 2 linear segments (Fig. 3b).

Based on the analysis, the radon gases observed in the 6 locations (ZZK, LTTC, BYC, CJA, NYC and WFS) were primarily from the first type (Fig. 2a). The gaseous radon observed in the other 7 locations (ZJY, YXZ, DYZ, NKC, XHZ, YHM and FN) should be a mix of the two species; the gas radon with concentration between T^1^ and T^2^ could be supplied by the first population, the origin of radon with concentration over T^2^ was dominated primarily by the second population. Those below T^1^, with much smaller concentrations (ranging from 0 to 3.19 kBq m^−3^) (Table 1), could be subjected to dilution by air through the conglomerate clay covering the 7 locations (Fig. 2c). The radon observed in the remaining 23 locations should be a mix of the two species too. The origin of radon with concentration over T^2^ was dominated by the second type (Fig. 2b), and a concentration under T^2^ was supplied by the first population.

**Figure 4:**
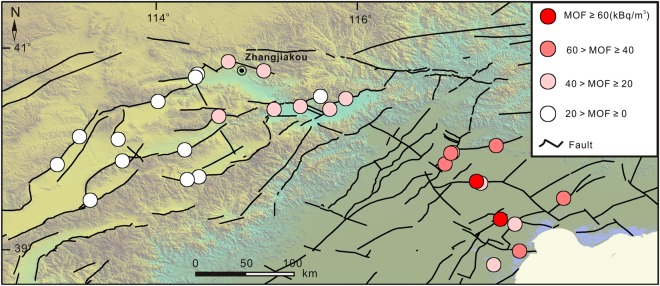
Spatial distribution of the mean values of fault-origin radon concentrations (MOF).

Fault-origin radon was observed in 30 locations (as described above), with a mean value of fault-origin radon concentrations (MOF) in the range of 7.65 kBq m^−3^ to 64.14 kBq m^−3^ (Table 1). Great spatial variations of MOF were observed across the study area (Fig. 4). Higher values of MOF were observed to the east of the Taihangshan piedmont fault zone in the Bohai Basin, in the range of 23.29 to 64.14 kBq m^−3^, which were significantly higher than those to the west of Taihangshan piedmont fault zone in the Basin and Range Province (7.65 to 32.11 kBq m^−3^) (Table 1).

**Figure 5:**
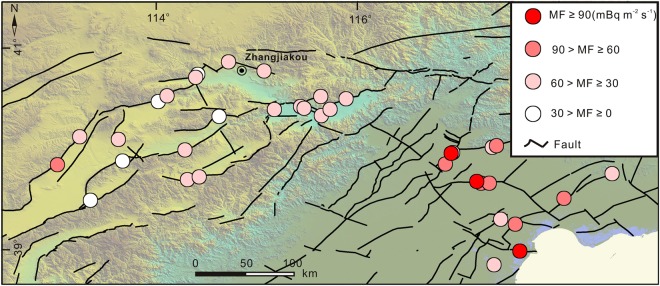
Spatial distribution of radon fluxes (MF) from faults.

**Figure 6:**
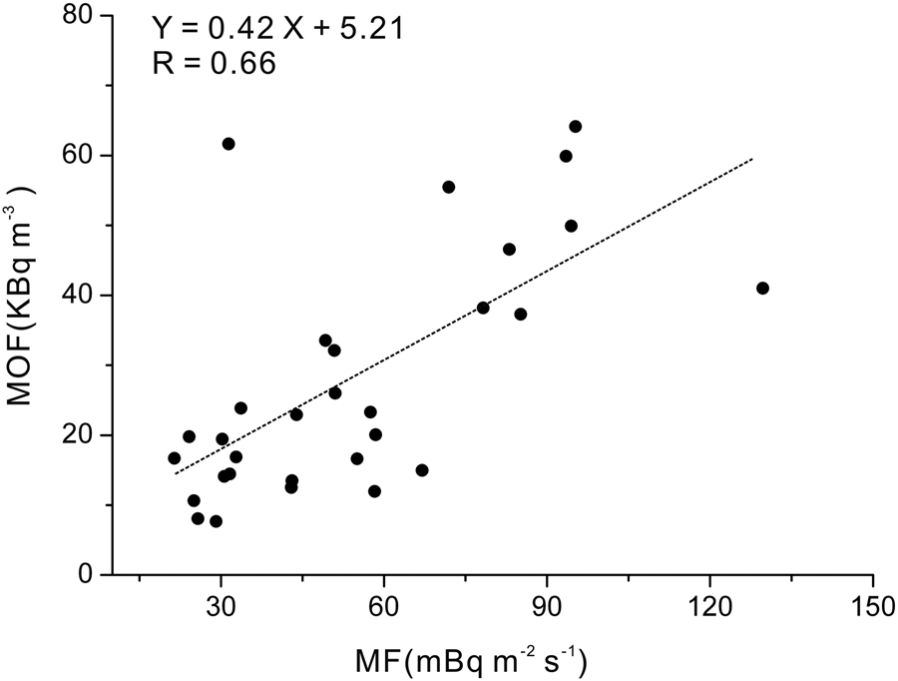
Plots of MOF versus MF from faults.

Radon fluxes measurements in 6 locations (ZZK, LTTC, BYC, CJA, NYC and WFS) with soil diffuse and disperse origins were carried out in the point with higher radon concentrations, and in the other 30 locations were performed where fault-origin radon was observed. The mean value of radon flux (MF) indicated a spatial distribution similar to that of MOF (Figs 4 and 5), with higher MF (31.43 to 129.74 mBq m^−2^ s^−1^) to the east of the Taihangshan piedmont fault zone in the Bohai basin compared (21.44 to 67.03 mBq m^−2^ s^−1^) to the west of Taihangshan piedmont fault zone in the Basin and Range Province (Table 2). In addition, the MF had a positive correlation with MOF (Y = 0.42x + 5.21, R = 0.66) (Fig. 6), suggesting that radon emitted from the locations where radon flux measurements had been performed could originate from radon migrating upwards through the faults and fractures.

**Figure 7:**
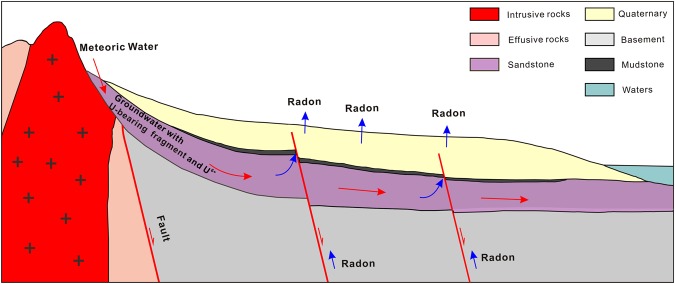
Model for the spatial distribution of radon concentrations in seismic zones near Beijing.

As a product of the natural radioactive decay of ^238^U^22^, the radon concentration in soil gas should correlate to the amount of underground ^238^U. However, sufficient ^238^U source in the Basin and Range Province areas around the basin and the wide gentle slopes in the boundary belt between the Basin and Range Province and basin could be favorable in accelerating the transfer of ^238^U from the Basin and Range Province to the basin where is accumulates^43^. In the study area, abundant intermediate-acid intrusive rocks containing uranium-bearing minerals were widely distributed around the basin (Fig. 1), with rugged hypsographic features to the west of the study area. When subjected to weathering, uranium-bearing rock fragments and dissolved uranium (U^6+^) could be transported from the boundary belt to the basin via syn-sedimentary groundwater, and accumulate in sandstone reservoirs (Fig. 7). Sandstone enriched with uranium has been reported in the Bohai Bay Basin^44^. Therefore, the radon generated from the decay of ^238^U accumulates in the sandstone reservoirs of the Bohai Bay Basin, escaping to the surface by upward migrating through faults, resulting in higher MOF and MF in the Bohai Bay Basin (Figs 4 and 5).

In addition, the high permeability of the fault zones could be another important factor contributing to the high concentrations and fluxes of gas emitted from active faults^26,45,46^. The permeability of the strata in the Bohai Bay basin could be higher than those in the Basin and Range Province, inferred by a higher Poisson’s ratio of the strata in the Bohai Bay Basin compared to that in the Basin and Range Province zone to the west of the Taihangshan piedmont fault zone^47^. Therefore, the uranium-bearing source in the west Basin and Range Province and higher permeability in the east basin area could be contributing factors for the spatial characteristics of radon concentration and fluxes observed in the Capital of China.

**Figure 8:**
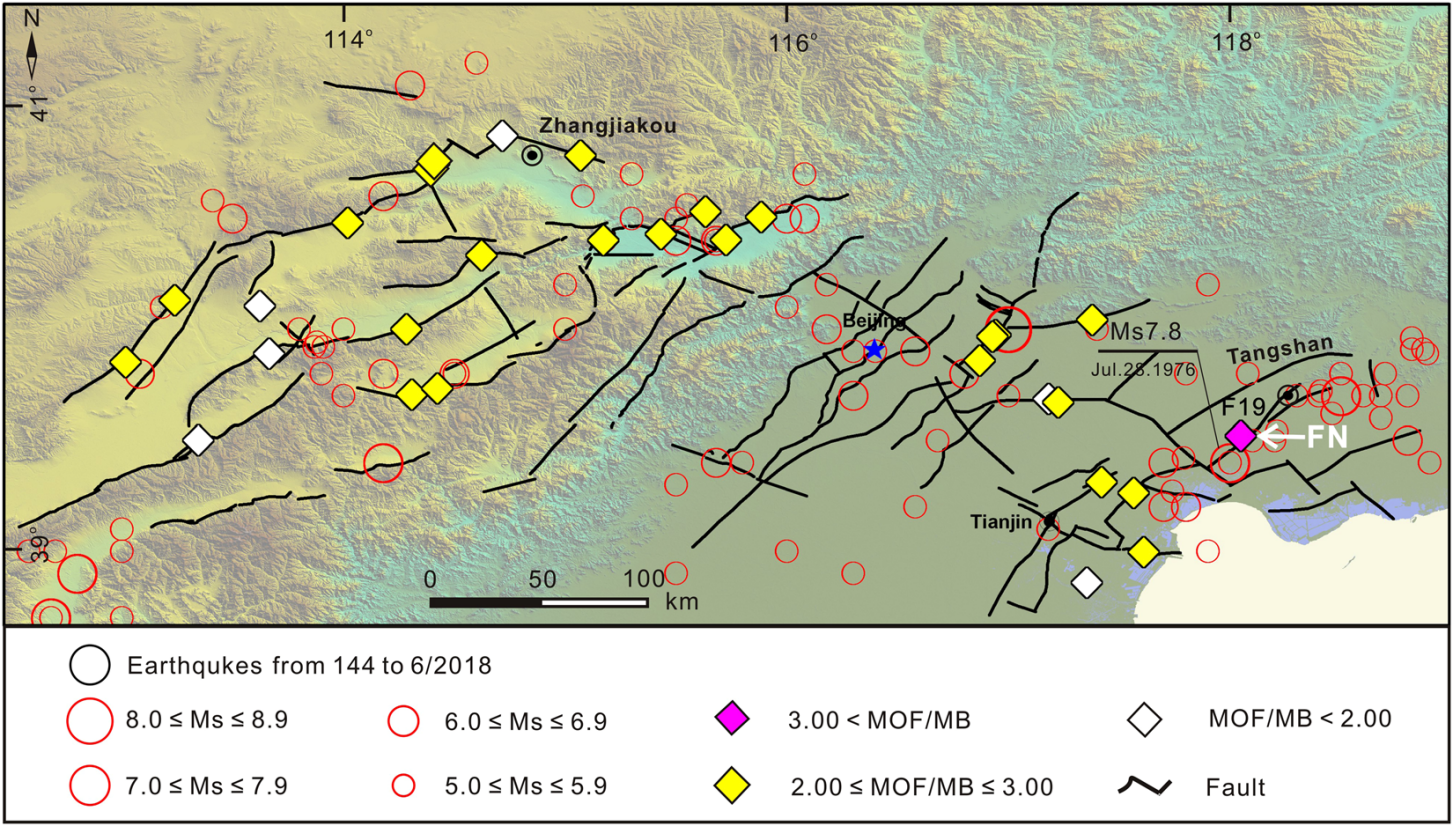
Spatial distribution of radon concentration intensities (MOF/MB) from faults.

Considering the difference in spatial distribution of MB (MB observed in the Bohai Bay Basin was 1.01 to 10.26 times higher than that in the Basin and Range Province to the west of the Taihangshan piedmont fault zone) (Table 1), the intensity of the radon concentration (MOF/MB) was calculated, and the relationship between MOF/MB from the faults and seismic activity in the study area was analyzed. The MOF/MB from the faults were 1.84 to 3.90, and the FN location along the TSF fault had the highest MOF/MB (3.90), with a high radon concentration and flux of 57.67 kBq m^−3^ and 334.56 mBq m^−2^ s^−1^, respectively (Tables 1 and 2, Fig. 8).

Previous studies report that strong earthquakes can enhance the radon degassing from deep in the earth through faults^26,48,49^. However, the water-rock interaction and transportation by groundwater, can result in clay minerals accumulating and clogging the fractures in the faults^38,50^, inhibiting the release of gas from the faults. Historically, 109 earthquakes with *M*_*S*_ ≥ 5.0 have occurred in the study area, and the Tangshan *M*_*S*_ 7.8 earthquake (28 July, 1976) was the strongest earthquake in the study area since 1680, which was followed by 4 aftershocks with *M*_*S*_ ≥ 6.0 and the epicenter located at the FN site along the TSF fault (https://earthquake.usgs.gov/earthquakes/search/). The highest MOF/MB value (3.90) was observed at the same FN site near the epicenter of the 28 July, 1976 Tangshan *M*_*S*_ 7.8 earthquake. Therefore, the radon degassing from the faults could have been enhanced by strong earthquakes over decades; 42 years in the case of the Tangshan *M*_*S*_ 7.8 earthquake.

As a natural and radioactive gas, the sudden and catastrophic, or quiet and continuous release of radon into the near-surface environment can result in health risks to the inhabitants living in adjacent areas^3,6,51^.

**Figure 9:**
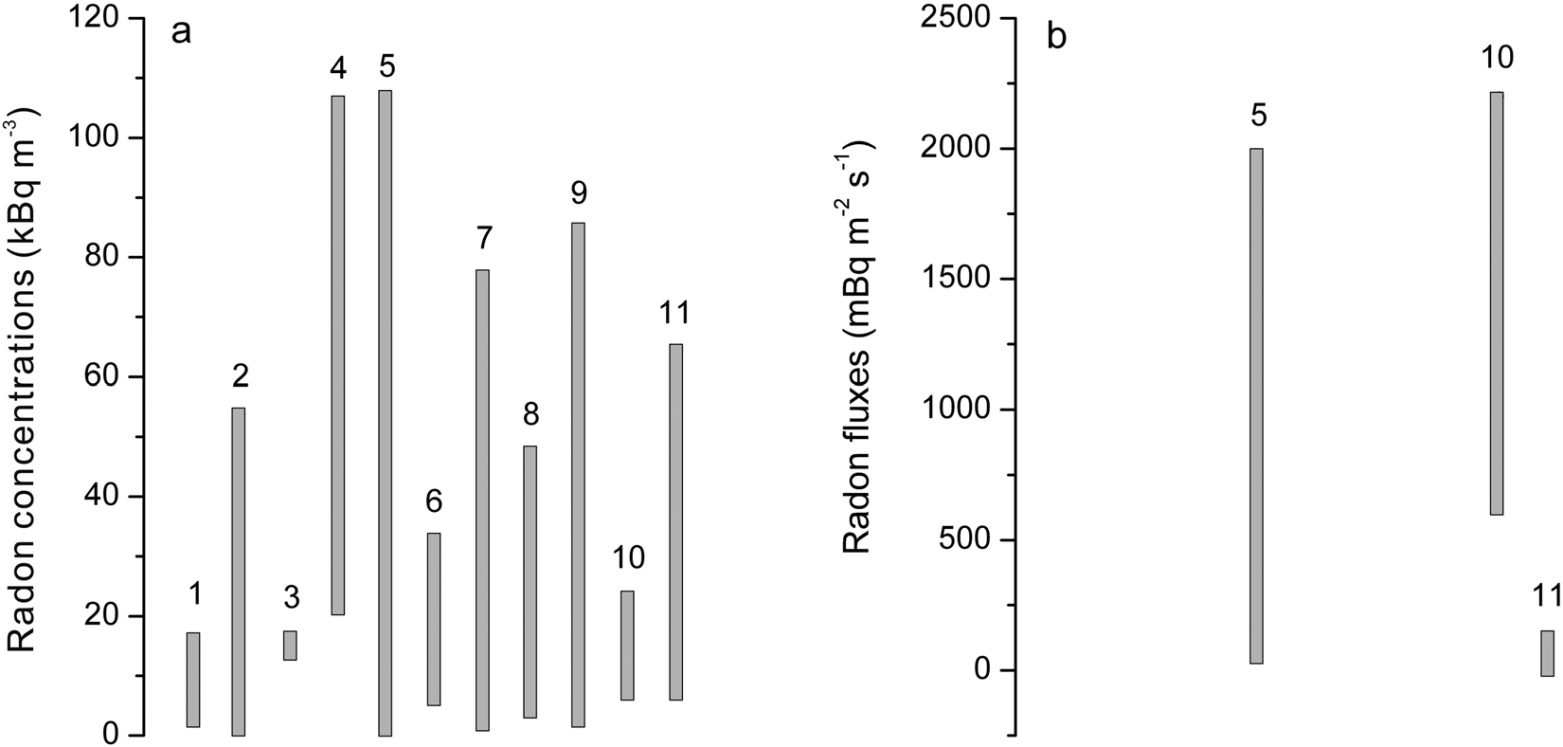
Concentration and fluxes of soil radon gas emitted from different sources. (**a**) concentration of soil radon gas, (**b**) fluxes of soil radon gas. Numbers above vertical thick lines correspond to the location/emission bodies listed in Table 3.

Higher soil radon concentrations and fluxes have been widely observed in fault zones^8,11,14^, due to (1) fault displacement during the late Quaternary Era, and (2) recent earthquakes in nearby faults^15,51^. In this study, non-negligible radon exhalations from active fault zones in the seismic zones near Beijing were observed (Tables 1 and 2), with soil radon concentrations of 7.65 to 64.14 kBq m^−3^, comparable to the concentrations of radon in other fault zones (0.40 to 76.00 kBq m^−3^), seismic ruptures produced by strong earthquakes (0.04 to 106.64 kBq m^−3^), sandstone-type uranium deposits (2.23 to 84.74 kBq m^−3^), and forests where nuclides were released accidentally from the Fukushima Daiichi Nuclear Power Plant in March 2011 (7.50 to 23.00 kBq m^−3^). Furthermore, soil radon fluxes from active fault zones in the seismic zones near Beijing were 21.44 to 129.74 mBq m^−2^ s^−1^, comparable to soil radon fluxes from seismic ruptures of the 2008 Wenchuan *M*_*S*_ 8.0 earthquake (45.90 to 1976.90 mBq m^−2^ s^−1^) and forests where nuclides were released accidentally from the Fukushima Daiichi Nuclear Power Plant in March 2011 (640.00 to 2200.00 mBq m^−2^ s^−1^) (Table 3, Fig. 9). Both the high radon concentrations and fluxes indicate that attention should be given to the environmental effects of radon emission from active fault zones in the seismic zones near Beijing, China.

**Table 3:** List of concentration and fluxes of soil radon gas from different sources globally.

No.	Location/Emission bodies	Radon concentration kBq m^−3^	Radon flux mBq m^−2^ s^−1^	References
1	China/Maqu fault	3.12~15.41	—	Zhao *et al*.^54^
2	Pyrenees/Amer fault	0.40~53.70	—	Zarroca *et al*.^55^
3	Italy/Timpe fault system	13.95~15.94	—	Vizzini and Brai^56^
4	Italy/Seismic rupture of the 1980, *M*S 6.9 Irpinia earthquake	22.00~106.00	—	Ciotoli *et al*.^7^
5	China/Seismic rupture of the 2008, Wenchuan *M*S 8.0 earthquake	0.04~106.64	45.90~1976.90	Zhou *et al*.^26^
6	Taiwan/Six major active faults in northern Taiwan	6.60~32.20	—	Fu *et al*.^57^
7	Pyrenees/Maladeta fault	2.00~76.00	—	Moreno *et al*.^6^
8	China/Sandstone-type uramum deposit in Wenjialiang region	4.48~47.21	—	Song *et al*.^58^
9	China/Sandstone-type uranium deposits in Erlian Basin	2.34~84.74	—	Liu *et al*.^59^
10	Fukushima/A forest where nuclides were released by the accident of Fukushima Daiichi Nuclear Power Plant in March 2011	7.50~23.00	640.00~2200.00	Fujiyoshi *et al*.^60^
11	China/Active fault zones in the capital area	7.65~64.14	21.44~129.74	This study

“—”: no data.

**Figure 10:**
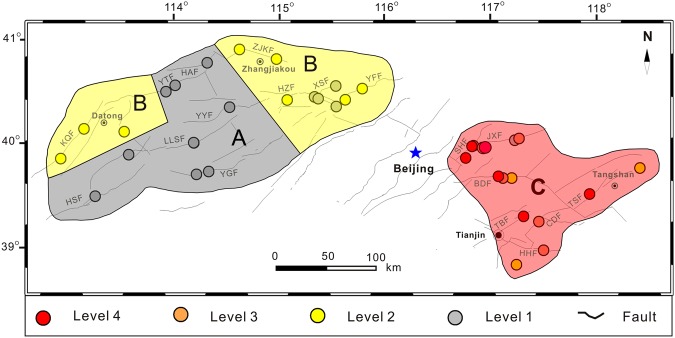
Identified zones in the study area with different amounts of radon pollution.

The Chinese code for indoor environmental pollution control of civil building engineering (GB 50325-2001, 2006) was used to divide the study area into 4 zones, including one “A”, two “B” and one “C” (Fig. 10). The “C” zone was the most highly radon polluted area from the faults in the study area, which included all the locations along the faults in the Bohai Bay Basin. The level of radon concentration and fluxes in 13 of the 14 locations within the Bohai Bay Basin were levels 2 to 4. Three locations (CJA, HJW and WFS) were level 2 only, 5 locations (QXZ, BHC, WZ, BHD and DSG) were level 3 and the other 5 locations (DDG, PGZ, WJKC, ZTD and FN) were level 4. The maximum soil radon concentration (97.55 kBq m^−3^) and flux (334.56 mBq m^−2^ s^−1^) were observed in ZTD and FN, respectively. Tables 1, 2 and 4 show the radon protective measures that should be required to protect the inhabitants from radon risk in buildings located along the faults in block “C” due to the levels of radon emitted from the faults. The two blocks “B” were meso-polluted areas caused by radon exhalation from the faults in the study area, which covered the locations distributed to the northwest and northeast of the Basin and Range Province, west of the Taihangshan piedmont fault zone (Fig. 10). The level of radon gaseous releases in 8 (CFY, WQX, QBK, HJP, YHM, SJC, SHZ and DTHS) out of 12 locations were level 2, although 4 locations (XHZ, DYZ, LTTC and BYC) in the northeast zone “B” were level 1 (Table 4), uniform radon protective measures should be recommended considering the close proximity to the 5 sites (CFY, WQX, QBK, HJP and YHM). Therefore, it is suggested that the underlying surface of the structure should be fixed in order to prevent cracking along the faults located in zone “B”. The zone “A” is a pollution-free area, which includes 10 locations (NCY, DHZK, YJY, YLK, ZZK, YJG, ZJY, NKC, YXZ and BKC) in the Basin and Range Province to the west of the Taihangshan piedmont fault zone (Fig. 10), where the levels of radon concentration and fluxes in all these locations were level 1, indicating that no pollution prevention need to be carried out up to now.

**Table 4:** Levels of radon emitted from the faults in the study area in accordance with the Code for Indoor Environmental Pollution Control of Civil Building Engineering in China (GB 50325–2010)^27^.

Levels	Radon concentration (kBq m^−3^)	Radon flux (mBq m^−2^ s^−1^)	Locations	Prevention regulations
1	0~20	0~50	NCY, DHZK, YJY, YLK, ZZK,YJG, ZJY, NKC, YXZ, BKC, XHZ, DYZ, LTTC, BYC, NYC	No need of pollution prevention and control of radon
2	20~30	50~100	CFY, WQX, QBK, HJP, YHM, CJA, HJW, WFS, SJC, SHZ, DTHS	The underlying surface of the structure should be fixed in order to prevent cracking
3	30~50	100~300	QXZ, BHC, WZ, BHD, DSG	Underlying surface of the structure should be remedied in order to prevent cracking, and water proof processing
4	>50	>300	DDG, PGZ, WJKC, ZTD, FN	Comprehensive prevention and control of radon pollution

## Conclusions

Radon in soil gas from two main sources in the active fault area in the capital of China was identified: (1) radon diffusing and dispersing from the permeable soil, and (2) radon upwelling from faults. Atmospheric dilution occurred in locations ZJY, YXZ, DYZ, NKC, XHZ, YHM and FN, as a result of air circulating through the conglomerate clay.

Spatial variations in radon concentration and fluxes across the study area were observed, including higher radon concentration and fluxes in the Bohai Bay Basin compared to those in the Basin and Range Province to the west of the Taihangshan piedmont fault zone. This phenomenon could be a result of the uranium accumulation in the sandstone reservoirs and the higher permeability of strata in the Bohai Bay Basin area.

The radon degassing from faults was enhanced by strong earthquakes, with the concentration of radon from the faults being from 1.84 to 3.90 kBq m^−3^. The highest MOF/MB value (3.90) was obtained at the FN site located in the epicenter zone of the Tangshan *M*_*S*_ 7.8 earthquake, with high radon concentration and flux of 57.67 kBq m^−3^ and 334.56 mBq m^−2^ s^−1^, respectively, being measured.

According to the Chinese Code for Indoor Environmental Pollution Control of Civil Building Engineering (GB 50325-2001, 2006), the Bohai Bay Basin is the most heavily radon polluted area by radon exhalation from the faults in the area, with the levels of radon concentrations and fluxes in 13 of 14 locations reaching levels 2 to 4, while the northwest and northeast zones of the Basin and Range Province to the west of the Taihangshan piedmont fault zone were meso-polluted, with levels of radon in 8 of 12 locations reaching level 2. It is suggested that corresponding radon protective measures should be in place to protect the inhabitants in buildings along the faults from emitted radon, and the release of radon in the active fault zones should be assessed to determine the possible risks.

## Methods

### Measurement design

The concentration and flux of radon in soil gases were measured in the field from 36 profiles, which were approximately perpendicular to the fault scarps. The profiles were repeated 3 to 9 times from May 2012 to September 2016. One or three parallel survey lines at each profile were measured. Sampling sites along survey lines were at intervals of 5 to 40 m, which were 5 m near the fault scarps and lengthening gradually to the ends of survey line away from the fault scarp, with a maximum interval distance of 40 m. The distance between the two neighboring measurement lines was 10 m. The concentration measurement was carried out at locations along the profiles across the fracture zones; the total number of the concentration values for each location ranged between 108 and 324, and the flux measurement was carried out at the locations in the fracture zones. The total number of the flux values for each location ranged between 12 and 36. In total, 5080 radon concentrations and 720 values of radon flux were measured in the study area.

### Measuring apparatus and procedure

**Figure 11:**
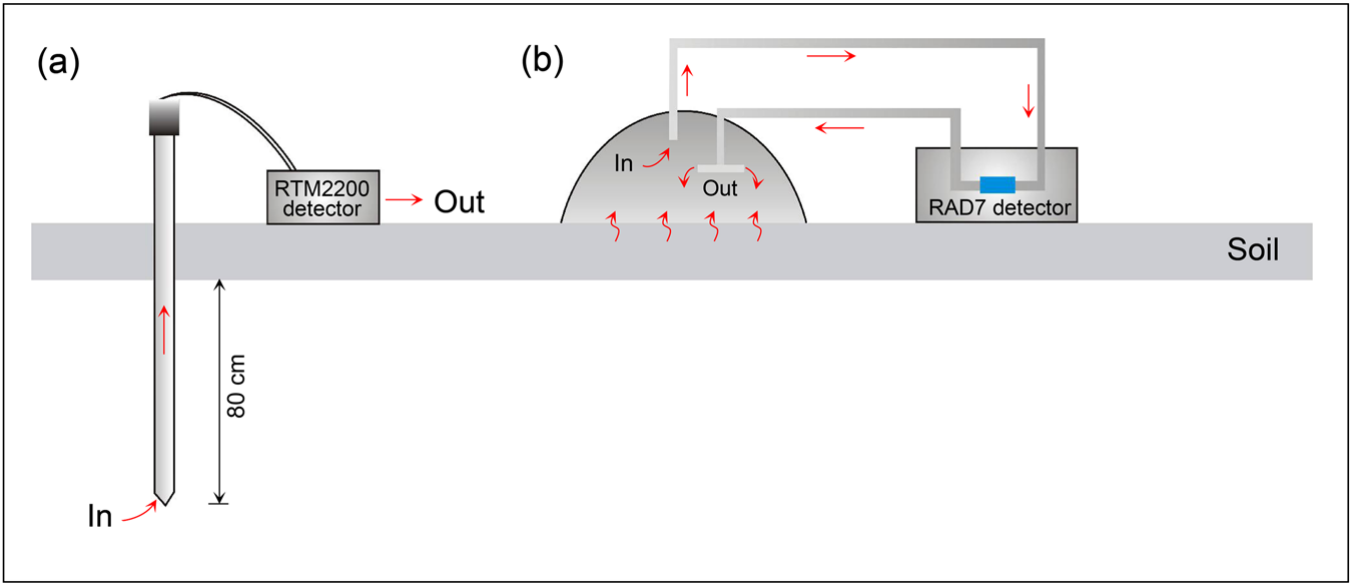
Sketches of measurement methods for radon concentration (**a**) and radon flux (**b**).

The mechanism of measurement using RAD 7 and RTM 2200 radon detectors was based on an energy spectrum analysis. ^222^Rn is an inert gaseous alpha-emitter with a half-life of 3.82 days. The nucleus of ^222^Rn decays along the sequence ^218^Po, ^214^Pb, ^214^Bi, ^214^Po, ^210^Pb, ^210^Bi, ^210^Po and ^206^Pb. With each transformation, the nucleus emits radiation (alpha and beta particles, or gamma rays) with a characteristic energy. ^218^Po has a half-life of 3.05 min, and decays by the emission of an alpha particle of 6.00 MeV. Due to its short half-life, a radioactive equilibrium can be achieved in 15 min, which reduces the background and improves the sensitivity of the apparatus. Thus, RAD 7 and RTM 2200 are designed to detect radon concentrations based on energy spectrum analysis using a solid-state detector as it decays to ^218^Po. The energy is transformed to an electrical signal, amplified and converted to digital information using electronic circuits. The radon concentration can be calculated from the accumulative decay information. Radon concentration was performed by inserting a stainless-steel sampling tube with a diameter of 3 cm into the ground to a depth of 80 cm (Fig. 11). The sampler was connected to the radon detector using a rubber tube. The radon concentration was measured in the field using a SARAD RTM 2200 radon detector (Fig. 11a). Radon values were obtained 15 min after measuring (time necessary to reach Po and Radon nuclei equilibrium, approximately 5 times the half-life of ^218^Po). An inlet filter and molecular sieve were used to protect the detector from dust and soil moisture (>10%). The detection limit and measurement error of the SARAD RTM 2200 were 500 Bq m^−3^ and ±5%, respectively.

The soil gas radon flux was measured using a static closed chamber method. The instrument contained an inverted circular accumulation hemispherical chamber, with a volume of 1.68 × 10^−2^ m^3^ and radius of 0.2 m, and a RAD7 radon monitor (with detection limit of 14.8 Bq m^−3^ and accuracy of ± 4%)^52^ (Fig. 11b). The gas circulated from the chamber to the monitor and then back into the chamber via a small-diameter plastic tube (3 mm inner diameter). In order to ensure the immediate and homogeneous mixture of the gas in the chamber, an 8-channel deconcentrator was installed onto the inner wall of the chamber to re-inject the circulating gas. The variation of radon concentration inside the chamber during flux measurements was recorded every 5 min.

### Calculation method

Radon flux (expressed as 10^−3^ Bq m^−2^ s^−1^) was calculated using the following Eq. (1):

1 $$Flu{x}_{Rn}(mBq{m}^{-2}{s}^{-1})=\frac{{\rm{\Delta }}C}{A{\rm{\Delta }}t}=\frac{{V}_{i}{P}_{i}{T}_{S}}{{P}_{S}{T}_{i}A}\cdot \frac{dc}{dt}$$

where: *Flux*_*Rn*_ (mBq m^−2^ s^−1^) is the soil radon flux, V_i_ (m^3^) and A (m^2^) are the volume and bottom area of the chamber, respectively. ΔC_Rn_ (Bq m^−3^) is the variation of radon concentration with time in the chamber during the measuring period Δt (min), *P*_*S*_ and *T*_*S*_ are the standard barometric pressure and temperature, *P*_*i*_ and *T*_*i*_ are the gas pressure and temperature inside the chamber.

### Statistical analysis

Kurtosis-Skewness test and Q-Q plots are usually used together to determine the species in the soil gas^35,46,53^. Statistical analyses for the collected data were subjected to the Kurtosis-Skewness test and Q-Q (normal quantile-quantile) plots. The data set with a normal distribution is usually from a single origin. Kurtosis-Skewness test was used to test the normal distribution for a data set; the ρ-values of a data set > 0.05 indicated that the data were of normal distributed. A Q-Q plots is effective in distinguishing different or overlapping species^18^. It revealed approximating linear segments (identifying gaps or inflection points) of a probability curve, a single linear distribution in the Q-Q plots indicating a normal distribution and single type for a data set, the points between different straight line segments indicated an abnormal distribution and different species for a data set, and the threshold values were determined using abscissa levels^34^.

## Data Availability

All data included in the manuscript are available upon request by contacting with the corresponding author.
